# Shell Disorder Models Detect That Omicron Has Harder Shells with Attenuation but Is Not a Descendant of the Wuhan-Hu-1 SARS-CoV-2

**DOI:** 10.3390/biom12050631

**Published:** 2022-04-25

**Authors:** Gerard Kian-Meng Goh, A. Keith Dunker, James A. Foster, Vladimir N. Uversky

**Affiliations:** 1 Goh’s BioComputing, 38 Hillside Drive, Singapore 548957, Singapore; 2 Center for Computational Biology and Bioinformatics, Indiana University School of Medicine, Indianapolis, IN 46202, USA; kedunker@iupui.edu; 3 Department of Biological Sciences, University of Idaho, Moscow, ID 83844, USA; foster@uidaho.edu; 4 Institute for Bioinformatics and Evolutionary Studies, University of Idaho, Moscow, ID 83844, USA; 5 Department of Molecular Medicine, Morsani College of Medicine, University of South Florida, Tampa, FL 33612, USA; vuversky@usf.edu; 6 Institute for Biological Instrumentation, Russian Academy of Sciences, Pushchino, 142290 Moscow Region, Russia

**Keywords:** coronavirus, COVID-19, intrinsic disorder, membrane, nucleocapsid, nucleoprotein, Omicron, pangolin, shell, virulence, spread, transmission, lung, bronchus, saliva, mucus, severe acute respiratory syndrome

## Abstract

Before the SARS-CoV-2 Omicron variant emergence, shell disorder models (SDM) suggested that an attenuated precursor from pangolins may have entered humans in 2017 or earlier. This was based on a shell disorder analysis of SARS-CoV-1/2 and pangolin-Cov-2017. The SDM suggests that Omicron is attenuated with almost identical N (inner shell) disorder as pangolin-CoV-2017 (N-PID (percentage of intrinsic disorder): 44.8% vs. 44.9%—lower than other variants). The outer shell disorder (M-PID) of Omicron is lower than that of other variants and pangolin-CoV-2017 (5.4% vs. 5.9%). COVID-19-related CoVs have the lowest M-PIDs (hardest outer shell) among all CoVs. This is likely to be responsible for the higher contagiousness of SARS-CoV-2 and Omicron, since hard outer shell protects the virion from salivary/mucosal antimicrobial enzymes. Phylogenetic study using M reveals that Omicron branched off from an ancestor of the Wuhan-Hu-1 strain closely related to pangolin-CoVs. M, being evolutionarily conserved in COVID-19, is most ideal for COVID-19 phylogenetic study. Omicron may have been hiding among burrowing animals (e.g., pangolins) that provide optimal evolutionary environments for attenuation and increase shell hardness, which is essential for fecal–oral–respiratory transmission via buried feces. Incoming data support SDM e.g., the presence of fewer infectious particles in the lungs than in the bronchi upon infection.

## 1. Introduction

### 1.1. COVID-19, SARS-CoV-2, and Omicron

Coronavirus disease 2019 (COVID-19) was first observed as an outbreak in Wuhan, China, in December 2019. COVID-19 is known to be often fatal, and many patients need oxygen ventilators to assist with breathing [1]. After the outbreak in Wuhan, COVID-19 rapidly spread throughout China and the rest of the world. The virus responsible for COVID-19 was named SARS-CoV-2 (severe acute respiratory syndrome coronavirus 2), as it has about 79% genetic similarity to the 2003 SARS-CoV (SARS-CoV-1) [1,2,3]. A retrospective search among the archives for close relatives of this virus yielded a genetic sequence from the RaTG13 sample of a beta-coronavirus isolated from bats found in Yunnan, China, with a 96% homology to SARS-CoV-2 [4,5]. Close relatives of SARS-CoV-2 were also found among Malayan pangolins: two sets of coronavirus (CoV) samples retrieved from smuggled pangolins that were confiscated in Guangdong and Guangxi, China, during 2019 and 2017/18, respectively. They have approximately 90% sequence identity with SARS-CoV-2 [6,7,8,9,10].

Since the first outbreak in December 2019, many variants have overtaken the parent Wuhan-Hu-1 strain (NCBI accession code NC_045512.2). Many of these variants were identified by their differences in their spike (S) glycoprotein. The first variant that was regarded as a variant of concern (VOC) by WHO was SARS-CoV-2 Alpha (B.1.1.7), which emerged in December 2020 and was 50% more contagious than the original Wuhan-Hu-1 strain [2]. It differs from the Wuhan-Hu-1 isolate by the presence of three mutations in the S protein: N501Y, D614G, and P681H. There were several WHO-designated subsequent VOCs, including the Beta (B.135) and Gamma (P1) variants. The most troublesome variants at the time of writing of this manuscript are Delta (B.1.1.617.2) and Omicron (B.1.1.529). Delta was first found in India around May 2020 and spread quickly around the world. It was found to be 50% more contagious than the Alpha variant and has several mutations not seen in previous variants, such as L452R, T478K, and P681R. Omicron came later with the first detection by South African physicians in November 2021 [1,11,12,13]. This heavily mutated variant was found to be less severe than previous variants, but approximately twice as infectious as Delta. There is, however, something else strange about Omicron. Many of the 50 mutations found in Omicron were never seen before in previous variants [14]. This essentially implies that this variant had been hiding somewhere for much of the pandemic. In fact, phylogenetic studies using S have traced the lineage to the Wuhan-Hu-1 strain dating back to February 2020 [14]. Our study will provide evidence that Omicron did not emerge as a descendant of the Wuhan-Hu-1 SARS-CoV-2, but is instead an ancestor of the Wuhan-Hu-1 isolate that is closer to pangolin CoVs.

The result, as we will see, arose from a phylogenetic study using M (membrane, outer shell) protein, a part of the CoV outer shell. A strange characteristic pertaining to the COVID-19 M proteins was first detected in a study during the pandemic using the shell disorder models (SDMs) [15,16,17]. It was discovered that among CoVs, all COVID-19-related viruses have relatively hard outer shells. A hard outer shell or low M-PID (percentage of intrinsic disorder) is associated with higher resistance of a virus to harsh conditions, which, in turn, reflects the mode of viral transmission. Hard shell disorder can be found in viruses infecting burrowing animals even if they are distantly related, as their fecal–oral transmission of CoVs is likely to involve contact with feces that have been buried for a long time [16,17].

### 1.2. The Three Closely Related Shell Disorder Models

The SDMs that were used to study CoVs consist of three closely related models [15,16,18,19,20,21,22,23,24]. The parent “viral shape shifter” model was built based on the observation that a large number of HIV-1 variants have unusually high disorder levels in the outer shell (matrix) that are not commonly found among viruses [15,16,20,23,24,25]. This characteristic is believed to be associated with the sexual transmission of viruses and the failure to find an effective vaccine for HIV, HCV, and HSV [15,20,26,27]. A daughter model was developed in 2012, when we published the transmission–shell disorder model, in which correlations between modes of transmission and shell disorder were described [22]. From 2015 onward, a series of publications provided the groundwork for a third model, the virulence–inner shell disorder model [15,18,19,20,21]. In this model, a strong relationship was found between the virulence and inner shell disorder for a variety of viruses, such as Ebola, Nipah, Zika, and Dengue.

### 1.3. The Shell Disorder Models as Applied to SARS-CoV-2

When the genetic information became available in the early stages of the SARS-CoV-2 pandemic, SDMs were readily deployed to understand the nature of this new and dangerous virus. SDMs use artificial intelligence (AI) to inspect the sequence of proteins and to predict disordered residues [28,29,30,31]. The PID provides a gauge for the level of disorder for a protein. According to the transmission–shell disorder model, SARS-CoV-2 fell into the same category as SARS-CoV-1 (2003 SARS-CoV), which has intermediate levels of potential for both fecal–oral and respiratory transmission [22]. This analysis was based mainly on the intermediate level of N-PID (i.e., nucleocapsid, inner shell protein), but there is something else very odd about SARS-CoV-2, which does not apply to SARS-CoV-1 or, for that matter, to most members of the CoV family. The outer shell (M) of SARS-CoV-1 is one of the hardest known, which is characterized by its low M-PID [16,17,32]. We believe that this is associated with the contagiousness of the virus. More specifically, the hardness of M provides greater virion protection against the antimicrobial enzymes found in the mucus and saliva. The hard M protein is conserved throughout all COVID-19 related viruses, including pangolin-CoVs [16,17,32].

### 1.4. Solving the Omicron Mystery Using Shell Disorder Models

With the emergence of Omicron as the current dominant SARS-CoV-2 strain, there are still many mysteries surrounding this variant as mentioned previously. Where did Omicron come from? Where was it hiding [14]? The SDMs might have specific answers to these questions. They have detected that the Omicron M is harder than M of any of the previous variants. The phylogenetic tree using M indicates that Omicron is a descendant of Wuhan-Hu-1, but arose from an ancestor of the Wuhan-Hu-1 isolate that is closer to the 2019 pangolin CoV. What is also stranger is that before the emergence of Omicron, the SDMs suggested that a precursor to SARS-CoV-2 entered humans in 2017 or before as an attenuated strain [16,17]. This analysis was based mainly on the N (nucleocapsid)-PID. When Omicron emerged, it was quickly determined clinically and experimentally to be milder than previous variants [12,13,33,34]. We found that the N-PIDs of both the pangolin-CoV 2017 and Omicron were nearly identical. Essentially, this means that the virulence–inner shell disorder model correctly predicted the attenuated nature of Omicron even before its actual emergence. We shall discuss the implications and evidence of these in greater detail.

## 2. Materials and Methods

An important concept used in SDMs is protein intrinsic disorder, which is defined as a lack of unique structure in the entire proteins or protein regions with specific biological functions [35,36,37,38,39,40,41]. Since there are several noticeable differences between the amino acid sequences of ordered and disordered proteins, multiple computational tools have been developed to recognize regions of disorder. One such tool is PONDR^®^ VLXT, which uses a neural network (artificial intelligence (AI)) trained using known ordered and disordered sequences to predict residues that are disordered [28,29,30]. PONDR^®^ VLXT is known to be highly sensitive to local sequence peculiarities and can efficiently find protein–protein interactions [42,43], making it suitable for the study of viral shell proteins, which are often ordered as a result of protein–protein complex formation. In fact, in the past, this algorithm has been successfully used to study a wide variety of viruses including HIV, Dengue viruses (DENV), Ebola (EBOV), Nipah, and SARS-CoV-1/2 [15,16,17,18,19,20,21,22,23,24,25,26,32,44,45,46,47]. PONDR-VLXT is freely available online [48].

The necessary protein sequences were retrieved from either UniProt [49] or NCBI-Protein/GenBank [50]. The obtained sequences were fed into the PONDR^®^ VLXT neural network. The resulting information and sequences were stored in a relational database using JAVA [51] and MYSQL [52]. The PONDR^®^ VLXT scores were also imported into OpenOffice spreadsheet [53], and the PONDR^®^ VLXT plots were created. The sequences downloaded were input into the NCBI BLASTP [54]. The graphical sequence comparison was used to study the sequence differences and was graphically adapted using GIMP [55]. The sequence similarity percentages were carefully noted and used as part of our analysis. Multivariate analyses were computed using R package [56] and the respective *r* (correlation) coefficients were recorded. Illustrations were drawn using both GIMP and OpenOffice.

The N and M sequences from a variety of CoVs were first uploaded separately onto EMBI-EBI website [57]. This CLUSTAL Omega software allows the generation of phylogenetic trees using M and N, respectively. The M sequences of COVID-19-related viruses were then uploaded into another website, TREX [58], that uses CLUSTALW to generate a phylogenetic tree using M with distance correction, unlike the trees generated by CLUSTAL OMEGA. The phylogenetic trees were enhanced and annotated using GIMP and used as part of the results.

## 3. Results

### 3.1. Mode of Transmission–Shell Disorder Model

The Mode of Transmission–Shell Disorder Model was developed 10 years ago with its first publication in 2012 [22]. The model categorized CoVs into three groups using M- and N-PIDs. Group A included CoVs with higher levels of respiratory but lower levels of fecal–oral transmission, group B contained viruses those with the intermediate potential for both fecal–oral and respiratory transmission, whereas group C encompassed CoVs with higher fecal–oral transmission potential and lower levels of respiratory transmission. When the initial paper was published in 2012 just before the emergence of the Middle Eastern Respiratory Syndrome (MERS), SARS-CoV-1 was found to be in group B [22], which consists of the CoVs with intermediate respiratory and fecal–oral transmission potentials, since in SARS-CoV-1, the N- and M-PIDs were evaluated at 50% and 9%, respectively. When the MERS-CoV sequence became available after the outbreak, it became clear that MERS-CoV fell into group C (lower respiratory, higher fecal–oral transmission, given that MERS-CoV N- and N-PIDs were 43% and 10%, respectively, as seen in Table 1 [45]. The greater fecal–oral transmission potential behavior of MERS-CoV was actually observed [15], thereby validating the model.

**Table 1:** Categorization of CoVs by N- and M-PIDs to predict levels of the respiratory and fecal-oral transmission (*p* < 0.001, r = 0.8, N = 32). The original model involved categorization mainly by N-PID, and group D was not included. The updated model integrates new knowledge arising from SARS-CoV-2 and Omicron.

Coronavirus	M-PID	UniProt (U) Genbank (G) Accession Code (M Proteins) ^a^	N-PID	UniProt (U) Genbank (G) Accession Code (N) ^a^	Group/Remarks
HCoV-229E IBV(Avian) ^c^	23 10	P15422 P69606	56 56	P15130 Q8JMI6	Group A Higher levels of respiratory transmission Lower levels of fecal–oral transmission
Bovine PEDV (Porcine) ^c^ Canine (Resp.) HCoV-OC43 SARS-CoV-1 HCoV-NL63 Bats ^b^	7.8 8 7 7 8.6 11 11.2 ± 5.3	P69704 P59771(U) A3E2F6(U) Q4VID2(U) P59596(U) Q6Q1R9(U) A3EXD6(U)	53.1 51.7 50.5 51 50.2 49 47.7 ± 0.9	Q8V432(U) Q07499(U) A3E2F7(U) P33469(U) P59595(U) Q6Q1R8(U) Q3LZX4(U)	Group B Intermediate levels of respiratory and fecal-oral transmission
MHV (Murine) ^c^ MERS-CoV TGEV (Porcine) ^c^ Canine (Ent.) HCoV-HKU1 ^d^	8 9.1 14 8 4.5	Q9JEB4(U) K0BU37(U) P09175(U) B8RIR2(U) Q14EA7(U)	46.8 44.7 44.3 42.41 40 37.4	P03416(U) K0BVN3(U) P04134(U) Q04700(U) Q0ZME3(U)	Group C Lower levels of respiratory transmission Higher levels of fecal–oral transmission
SARS-CoV-2 [Wuhan-Hu-1] [Delta] [Omicron] Pangolin-CoV ^e^ Rabbit-CoV RaTG13	5.9 5.9 5.4 5.6 ± 0.9 5.7 4.1	YP009724393(G) QUX81285(G) UFO59282(G) QIA428617(G) H9AA37(U) QHT63303(G)	48.2 47.1 ± 0.5 44.8 46.6 ± 1.6 52.2 6 ^e^ 48.5	YP009724397(G) QYM89845(G) UFO692871(G) QIA48630(G) H9AA59(U) QHR63308(G)	Group D High respiratory and fecal–oral transmission potential

^a^ UniProt (U) [49]; GenBank-NCBI (G): [50] ^b^ Summary figures on bats. Further details on the bat samples can be found in Table 2. Four out of five bat-CoVs are in group B. High standard deviations are seen for N- and M-PIDs as denoted by ‘±’. (r = 0.8, *p* < 0.05, N = 32). ^c^ MHV (murine hepatitis virus), IBV (infectious bronchitis virus), PEDV (porcine epidemic diarrhea virus), TGEV (Transmissible, gastroenteritis virus). ^d^ HCoV-HKU1 has one of the lowest M-PID, which could qualify it for group D, but its N-PID is also abnormally low. Much is still not understood about HCoV-HKU1 [59,60]. For these reasons, HCoV-HKU1 remains in group C. ^e^ Details on the existing pangolin-CoVs known can be found in Table 2.

**Table 2:** Details of the pangolin CoVs and Bat CoVs N/M PIDs and their sequence similarities with the SARS-Co-V and SARS-CoV-2 as references. Using N-PID, SDMs have detected two sub-variants of Delta not previously detected (Delta1, Delta2).

Coronavirus	Sequence Similarity M (%)	M PID (%)	Accession: UniProt (U) GenBank (G)	Sequence Similarity N (%)	N PID (%)	Accession UniProt (U) GenBank (G)
SARS-CoV-1 Civet-SARS-CoV	90.5 90.1	8.6 8.6	P59596(U) Q3ZTE9(U)	90.5 90.01	50.2 49.1	P59595(U) Q3ZTE4(U)
Pangolin-CoV 2019 2018 2017 **	98.2 97.7 98.2	5.6 ± 0.9 ^a^ 6.3 4.5 5.9	QIG55948(G) QIQ54051(G) QIA48617(G)	98 93.8 94 93.32	46.6 ± 1.6 ^a^ 48.7 46.3 44.9 46.5	QIG55953(G) QIQ54056(G) QIA48630(G) QIA48656(G)
SARS-CoV-2 [Wuhan-Hu-1] [Delta1] [Delta2] [Omicron] **	100 99.1 99.1 98.7	5.9 5.9 5.9 5.4	YP009724393(G) QUX81285(G) QUX81285(G) UFO59282(G)	100 99.3 99.1 98.6	48.2 46.8 47.5 44.8	YP009724397(G) QYM89997(G) QYM89845(G) UFO692871(G)
Bat-CoV RATG13 Bat 512 HKU3 HKU4 HKU5	99.6 35.5 91 42.7 44.7	11.2 ± 15 ^a^ 4.1 15.3 7.7 16.4 11.8	Q9JEB4 QHR63303(G) Q0Q463(U) Q3LZX9(U) A3EXA0(U) A3EXD6(U)	99.1 29.4 89.6 51.1 47.9	47.7 ± 0.9 ^a^ 48.5 46.5 48 48.5 47.1	QHR63308(G) Q0Q462(U) Q3LZX4(U) A3EXA1(U) A3EXD7(U)

^a^ Standard deviation is denoted by “±”. ** Attenuated strains detected.

### 3.2. Hardest Outer Shell (Lowest M-PIDs) Is Seen in All COVID-19-Related CoVs: Burrowing Animals

**Figure 1:**
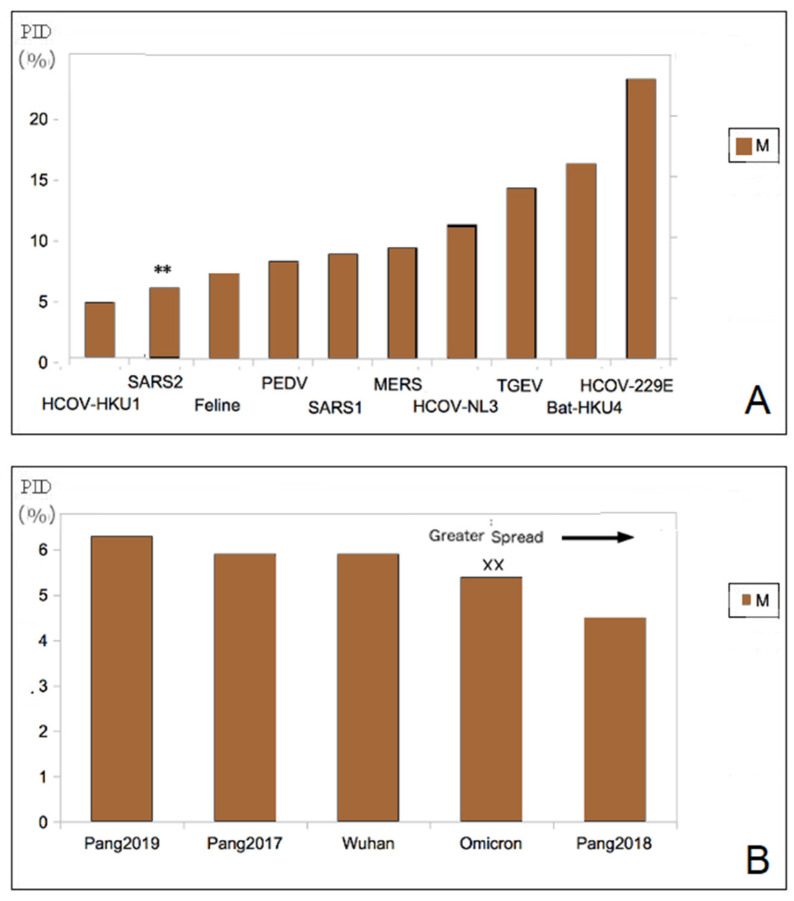
(**A**). All CoVs are closely related to SARS-CoV-2 (**). SARS-CoV-2 has the lowest M-PID (hardest outer shell) among all the CoVs analyzed in this study. (**B**). A hard outer shell is seen among COVID-related CoVs. Omicron (XX) has the lowest among all SARS-CoV-2 variants.

Yet another chance to validate the model came with the COVID-19 pandemic. This time, SARS-CoV-2 (Wuhan-Hu-1) (N PID: 48%) had to be placed in group B at that time alongside SARS-CoV-1 (N PID: 50%) [32]. While it does seem to validate the model, something that is very strange was also seen. SARS-CoV-2 (M PID: 5.8%) has one of the hardest outer shells in its entire CoV family (Figure 1) [32,47]. One exception is the rabbit-CoV (M PID: 5.7%), which is not closely related to SARS-CoV-2. Both rabbits and pangolins are burrowing animals [16,17]. Pangolin CoVs (M PID: 5.6 ± 0.9) have comparatively hard M and were found to be closely related to SARS-CoV-2 [16,17].

We will notice that, in Figure 1B, only HCoV-HKU1 has a harder M (M PID: 4.7%) than SARS-CoV-2. HCoV-HKU1 that was first disordered in a 71-year-old patient in 2004, is enigmatic and remains shrouded in mystery even today with respect to its evolution and epidemiology [59,60]. We have no idea how it entered the human population or its actual zoonotic source. Interestingly though, we do know from phylogenetic studies that it is closely related to murine-CoV, particularly MHV, which is itself enigmatic because rats and mice have evolved to live in burrows and human homes depending on the species involved. Our disorder data do actually reflect the enigmas. A more in-depth discussion of HCoV-HKU1 will be presented later.

### 3.3. High Infectivity of SARS-CoV-2 Is Related to Its Abnormally Hard Outer Shell (Lowest M-PIDs)

The original model was designed using knowledge of CoVs from farm animals, especially those of porcine. Strong correlations between the transmission mode and especially N-PID were observed (r^2^ = 0.77). A minute but statistically significantly greater correlation could be found when M-PID was added as an additional independent variable (r^2^ = 0.83). Biologically, inner shell proteins are known to assist the replication and disorder in the inner shell does enhance the replication [61]. It is for this reason, the correlations between virulence and inner shell disorder have been detected in a sister shell disorder model [18,19,20,21]. Similarly, greater disorder of the N protein allows greater copies to be present in vital organs, mucus, and saliva (saliva and mucus are more complicated as we will see). N disorder is therefore correlated with the mode of transmission potentials because of a minimal amount of infectious particles need to be present in the saliva and mucus before a virus can be transmissible by the respiratory modes [32].

M protein, however, is more enigmatic. Some viruses that have high fecal-oral transmission potentials do not need to remain in the environment for a long time, since fecal-oral transmission is often very efficient in farm animal, such as pigs, as in the case of the TGEV. It is for this reason that TGEV M-PID is somewhat high, at about 14%. While the slight correlation between M-PID and mode of transmission was detected, the exact nature of this relationship was impossible to understand, because there were very few CoVs with extremely low M-PIDs, as seen in Figure 1A [22,32,45]. The COVID-19 pandemic is actually an event that sheds light on the role of M in transmissibility. The unusually hard outer shell (low M-PID) can be seen among all of the SARS-CoV-2 close relatives, as shown in Figure 1 and Table 2. The high infectivity of SARS-CoV-2 is likely the result of its hard M protein protecting the virion from damage arising from the antimicrobial enzymes found in mucus and saliva [32,62,63]. In fact, hard outer shells are often found in other viruses that are commonly found in the saliva, such as rabies and DENV, which is exposed to mosquito saliva [19,25].

The emergence of the Omicron variant does not only provide even more proof of the role of M, but also highlights the close relationship of a harder M to transmissibility. Omicron is more infectious than the original Wuhan-Hu-1 isolate, or, for that matter, all previous SARS-CoV-2 variants, and it is also more infectious and has a harder M (M-PID, 5.4%) than previous variants including the Wuhan-Hu-1 reference isolate (M-PID, 5.9%) as seen in Table 1 and Table 2 and Figure 1. While we believe that the original transmission–shell disorder model is valid as a tool to gain insight to the evolution of CoVs, SARS-CoV-2 variants, particularly Omicron, have hinted that the model may not be complete, as the original model was designed without sufficient samples of CoVs with low M-PIDs. CoVs with extremely low M-PIDs should be categorized as a totally different group, i.e., group D, which consists of viruses with high fecal–oral and respiratory transmission, as seen in Table 1. This added feature is still able to maintain a good correlation (r^2^ = 0.7, *p* < 0.05).

**Figure 2:**
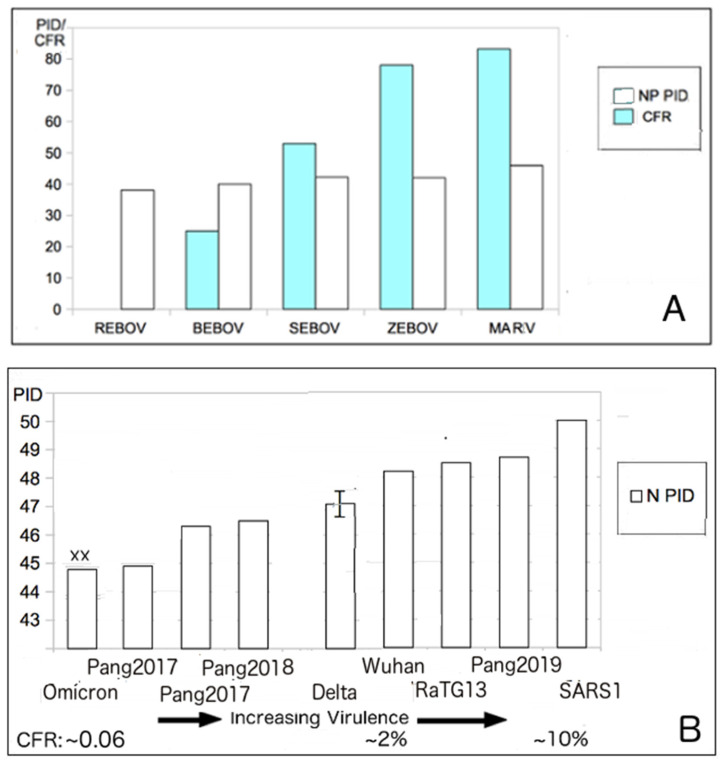
The virulence–inner shell disorder model. (**A**). Correlation of the Ebola (EBOV) virulence and inner shell (NP) disorder (r = 0.95, *p* < 0.05) [21]. (**B**). Correlation between SARS virulence and the inner shell (N) disorder (r = 0.8, *p* < 0.05). Abbreviations: Pangolin-CoV 2017–2019 (Pang2017–Pang2019). SARS-CoV-1 (SARS1), Wuhan (Wuhan-Hu-1), Reston EBOV (REBOV), Bundibugyo (BEBOV), Sudan (SEBOV), Zaire (ZEBOV), Marburg virus (MARV). “XX” denotes the Omicron variant.

Table 1 and Table 2 show that the M- and N-PIDs of Omicron (M-PID: 5.4%, N-PID: 44.8%) are different from those of Delta (M-PID: 5.9%, N-PID: 47.1 ± 0.5%) or Wuhan-Hu-1 reference isolate (M-PID: 5.9%, N-PID: 48.2%). While we have explained the significance of a lower M-PID, the disorder status of N tells a different story and is analyzed using the virulence–inner shell disorder model. The model is best summarized by Figure 2A. As in the case of Ebola virus, there is a correlation between the level of disorder of the inner shell protein and virulence [21]. As aforementioned, this correlation has been found in a variety of viruses, such as EBOV, DENV, and NiV [18,19,20,21]. The reason that the inner shell disorder is correlated with virulence has to do with the fact that the inner shell of viruses usually play important roles in replication. As a result, the higher disorder of the proteins in the inner shell provides means for more efficient viral replications. Protein intrinsic disorder defines partner pliability and determines better fits in protein–protein/DNA/RNA/lipid interactions [61,64,65,66,67,68,69,70,71,72,73,74,75,76,77,78,79,80,81,82,83,84,85,86,87]. Viruses often use the strategy of “Trojan Horse” for immune evasion [15,26,46,88], in which the virus would replicate very rapidly before the host immune system can detect its presence. This strategy can be deployed using greater disorder at the level of inner shell proteins. However, this strategy often backfires on the virus by overwhelming vital organs with a large number of virus copies and therefore killing the host.

### 3.4. SDMs Suggest That an Attenuated Precursor from Pangolins Entered Humans in 2017 or Earlier: Omicron Resembles Pangolin-CoV 2017 with Lower M-PID

Evidence that the virulence–inner shell disorder model is applicable to the SARS-CoV-1 and SARS-CoV-2 can be found in Figure 2B, showing a reasonably good correlation (r = 0.8. *p* < 0.05) between the case fatality rate (CFR) and N-PID. The CFRs of the various SARS-CoV-2 are estimated extrapolations [89], even if it is certain that the CFR of Omicron (0.16) [90] is certainly much lower than that of the other variants [90]. There is no evidence that the death rate of Delta is higher than that of the Wuhan-Hu-1 strain. The CFR estimates of the Wuhan-Hu-1 strain range from 0.5–2% [89] depending on the investigation but are definitely considered much higher than that of Omicron [3,13,90]. SARS-CoV-1 has a CFR of about 10% [3], which is definitely well above that of the SARS-CoV-2.

We will note that the virulence–inner shell disorder model predicts that Omicron and Pangolin-CoVs (especially pangolin-CoV 2017), are attenuated. Before the emergence of Omicron and upon the analysis of the pangolin CoVs, SARS-CoV-1, and SARS-CoV-2, the SDMs suggested that a precursor from pangolin-CoV may have entered humans and spread silently, as early as 2017 or earlier, as an attenuated virus before becoming more virulent.

This hypothesis was based mainly on the analysis of the N-PIDs. Upon the emergence of Omicron, it was quickly determined that this lineage is milder than that of the other SARS-CoV-2 variants. Within the frames of the typical SDM reproducibility, the N-PIDs of Omicron (44.8%) and pangolin CoV 2017 (44.9%) are almost identical (see below and Table 1 and Table 2). There was an assumption that the spread of the attenuated precursor could involve a slower spread, but this was based on the hypothesis that the M-PID of the precursor was the same as the M-PID of SARS-CoV-2 or higher. The pangolin CoV 2017 has the same M-PID as SARS-CoV-2, but Omicron has a lower M-PID (5.4%), which could account for its greater infectivity.

### 3.5. N Disorder Patterns: Pangolin CoV and Omicron and Wuhan-Hu-1 SARS-CoV-2

**Figure 3:**
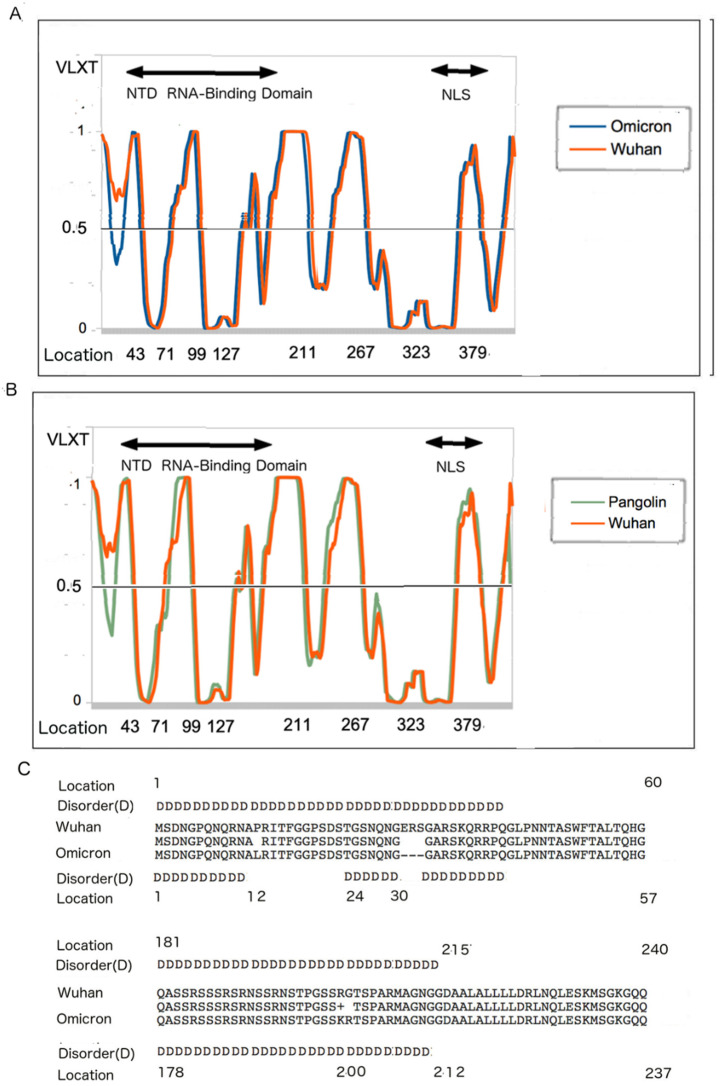
The PONDR^®^ VLXT plots and sequence peculiarities of different CoVs. Residues with the PONDR^®^ VLXT scores of 0.5 and above are considered disordered. (**A**) PONDR^®^ VLXT comparison of the N proteins the Wuhan-Hu-1 isolate and Omicron variant. (**B**) Comparison between the Wuhan-Hu-1 isolate and pangolin CoV 2017. (**C**) Sequence and disorder comparison of N proteins from the Wuhan-Hu-1 isolate and Omicron variant. Abbreviations: N-terminus RNA-Binding Domain (NTD RNA-Binding), Nuclear Localization Signal (NLS) [91].

Since PONDR^®^ VLXT is dependent on the protein sequence for its prediction, it is necessary to examine the sequence and disorder comparatively. Figure 3 shows the PONDR plots for N. Figure 3A represents a comparison of the PONDR plots of the N protein of the Wuhan-Hu-1 and pangolin strains, whereas Figure 3B compares Wuhan-Hu-1 and Omicron. While it has already been seen that Omicron N-PID (44.7%) resembles that of Pangolin CoV 2017 (44.8%) (see Figure 2B and Table 2), Figure 3 shows that the PONDR plots of the Pangolin CoV and Omicron resemble each other, when compared to the Wuhan-Hu-1 strain. Within the disordered N-terminal domains, the regions of local order are found around residues 12–23 of both Pangolin CoV and Omicron, but not in the Wuhan-Hu-1 isolate. The mutation (P13L) and deletions (31–33, ERS) could account for the difference in the disorder at the 12–23 region. The deletions at 31–33, which involve the deletion of polar residues (ERS), account for the fact that the N of Omicron is 416 AA long, whereas the N of the Wuhan-Hu-1 isolate has 419 residues.

### 3.6. Disorder Differences near the NTD RNA-Binding Region

Another noticeable difference in the disorder profiles can be found in Figure 3A, where there is a dissimilarity around locations 212–215, with the ordered region starting at 212 and 215 for the Omicron variant and Wuhan-Hu-1 reference strain (Figure 3C). This difference can be traced to both the differences in location as the result of the deletion at locations 31–33 and the mutations at R203K and G204R. Mutations around this region have been observed to induce virulence presumably by enhancing viral replication. It should be noted that deletion of the polar residues and mutations to less polar residues are often related to the induction of some local order [29,31,92,93,94,95,96], which is mostly the case for Omicron. It can be seen that most of the mutations leading to lesser disorder in Omicron and pangolin CoVs are located close to or within the NTD RNA-binding domain [91], which implies that the attenuation emerged from the inability of the two attenuated viruses to bind to the viral RNA more efficiently during the replication process.

### 3.7. The Phylogenetic Tree Using M (Membrane) Protein Provides the Greatest Accuracy as it Is Evolutionarily Conserved and Recombinations Can Confuse Previous Phylogenetic Studies

We have argued in previous papers that phylogenetic studies offer that best study as all viruses closely related to SARS-CoV-2 have the hardest outer shell (M protein) among its CoV family. From the sequence identities, especially COVID-19-related viruses, shows us that the M (membrane) proteins are among the most conserved proteins among COVID-19-related viruses. The evolutionary necessity of a hard outer shell can be found in their relationship with the burrowing pangolins and their fecal–oral–respiratory transmission via buried feces. This can be confirmed by data summarized in Table 2, with high sequence similarities among all the COVID-19-related viruses. Furthermore, reported studies argued that the use of a protein that is not conserved among strains and variants could lead to the mistake, as recombination could have happened, and the current phylogenetic algorithms handle recombination very poorly that could lead to generation of the wrong results [97].

### 3.8. The Phylogenetic Tree Using M Suggests That Omicron Did Not Arise from the Wuhan-Hu-1 Strain but from One of Its Ancestors That Are Closer to Pangolin CoVs

**Figure 4:**
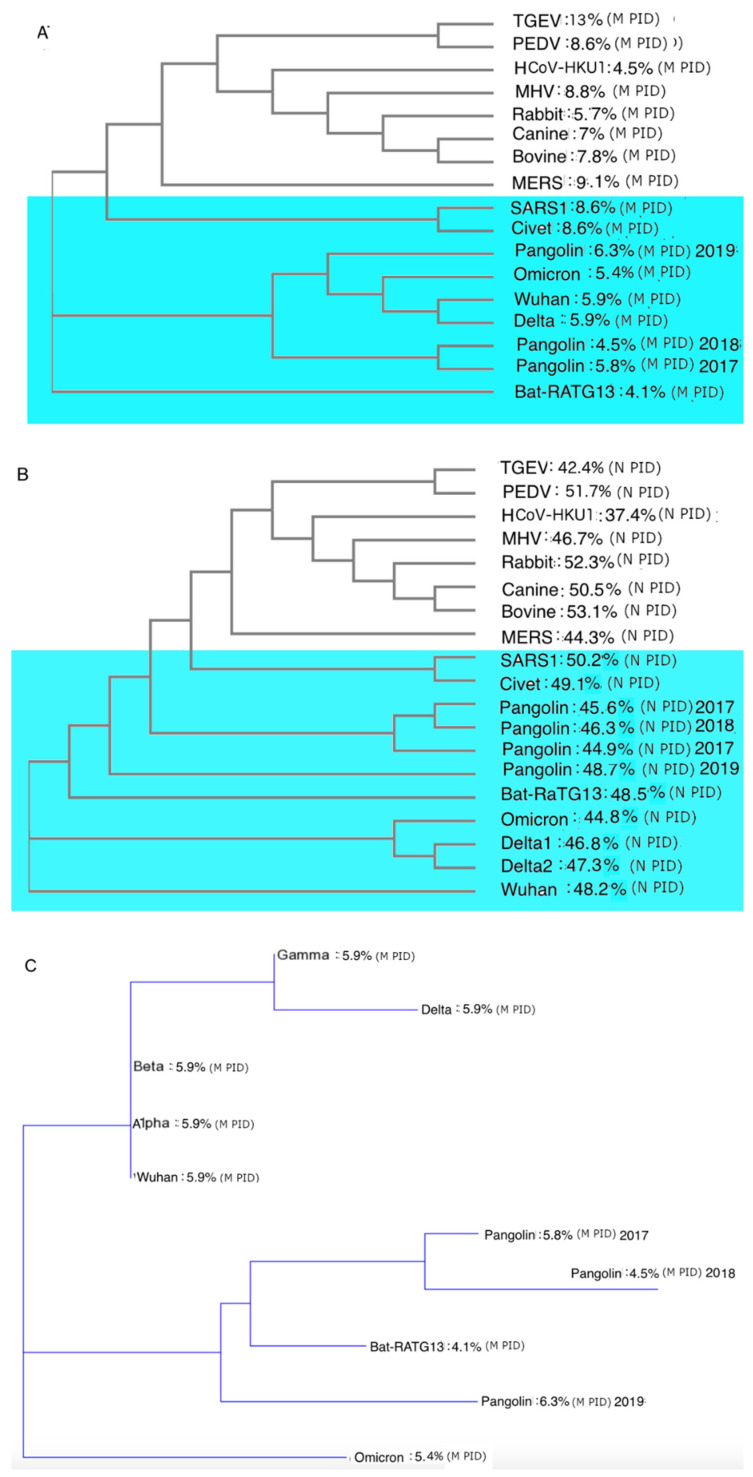
Phylogenetic trees using M (membrane) and N (nucleocapsid) proteins. (**A**) Phylogenetic tree using M protein. (**B**) Phylogenetic tree using N protein. (**C**) Phylogenetic tree using M protein for COVID-19-related variants and strains showing Omicron to be an entirely different branch from the other SARS-CoV-2 variants, and closer to pangolin CoVs. (Additional NCBI accession codes for M proteins: QXY06216 (Alpha), QWA53378 (Beta), and QWW7597 (Gamma). The phylogenetic tree in (**C**) includes distance correction, unlike those in (**A**,**B**).

The difference between phylogenetic trees of M and N can be found in Figure 4. As already seen in our previous studies, the phylogenetic tree using M clusters the pangolin CoVs near SARS-CoV-2, unlike those using other proteins or building a phylogenetic tree based on the genome-wide analysis. With Omicron, it yields further interesting and peculiar results. The phylogenetic tree using N (Figure 4A) shows us that Omicron probably split from the Wuhan-Hu-1 strain just as Delta and the other variants did. An examination of Figure 4A reveals that the split occurred earlier than that for Delta. This result is similar to the phylogenetic tree using S1 that tells us that Omicron, unlike other SARS-CoV-2 variants, split off from the Wuhan-Hu-1 strain within 1–3 months of the initial Wuhan outbreak. The phylogenetic tree using N seen in Figure 4B seems to be consistent with the phylogenetic tree using S1. The phylogenetic trees based on M, as seen in Figure 4A,C, indicate something else that is strangely different. This analysis suggested that Omicron did not descend from the Wuhan-Hu-1 strain, but rather originates from one of its ancestral strains. This is more clearly seen in Figure 4C, showing that the Omicron is closer to the pangolin CoVs than to the original Wuhan-Hu-1 strain or any other SARS-CoV-2 variants. This immediately raises many questions, which we will address later.

We also argue that the phylogenetic tree using M is the most accurate way of evolutionary analysis, as M is the most conserved, as seen in the consistently low M-PIDs among all COVID-19-related CoVs (Figure 4C), and there are high similarity levels among close relatives of SARS-CoV-2 (Table 2). High conservation means that there is lower likelihood of a recombination taking place for the protein and current phylogenetic algorithms handle such recombination poorly [97].

Figure 4C shows that the story remains consistent even when other VOCs are added (Alpha, Beta, and Gamma). We can observe that the M-PIDs (5.9%) of Alpha, Beta, and Gamma are identical to those of the Delta variant and the Wuhan-Hu-1 isolate. Omicron stands out alone at 5.4%. This has implications on the infectivity of Omicron as a harder outer shell will enable the virus to shed more particles nasally and orally as it will be more protected against the antimicrobial enzymes found in the saliva and mucus. How did Omicron manage to get these unique characteristics? We will discuss this later in more detail.

### 3.9. Omicron May Have Been the Result of a Reverse Zoonotic Transfer from Humans Back to a Burrowing Animal

**Figure 5:**
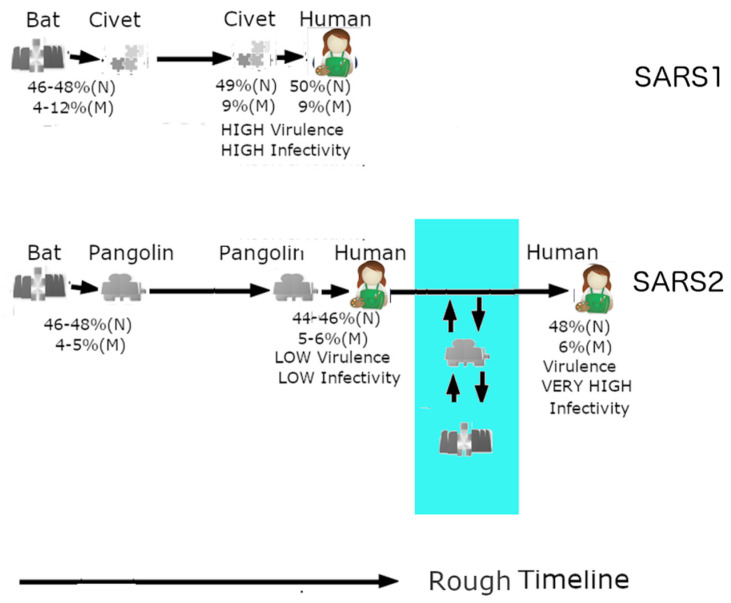
Zoonotic relationships of SARS-CoV-1/2. SARS-CoV-1 (SARS1) was likely in an intermediary host (palm civet cat) for a relatively short period, which could account for its high virulence. SARS-CoV-2 (SARS2), on the other hand, could have been in pangolins for a long period of time and thus first entered as an attenuated strain. Omicron had the opportunity to retain its attenuation by a reverse zoonotic transfer back to a burrowing animal (in blue).

From Figure 4A,C, we can see that pangolin CoVs cluster around SARS-CoV-2, making pangolin CoVs the closest relatives to SARS-CoV-2. Figure 5 summarizes the likely relationship that the SARS-CoV-1 and SARS-CoV-2 have with humans, bats, and covet cat/pangolins. Figure 5A shows the relationship between bat, virus, and civet cat. A bat version of SARS-CoV-1 had crossed into the palm civet cat and not long after that the civet cat virus entered the human population through human contact with civet cats. This took place in a relatively short period of time compared to SARS-CoV-2 in Figure 5. This prolonged virus–human interaction may have been assisted and preceded by a similarly prolonged period of interactions between the virus and pangolin. As for Omicron, it could have been assisted by a reverse zoonotic viral transfer from human back to pangolins or a similar burrowing animal as illustrated in the area shaded blue in Figure 5. It should also be noted that bats often cohabits with pangolins in burrows, which may provide an ideal environment for the exchange of viruses [98].

### 3.10. Life Cycles

**Figure 6:**
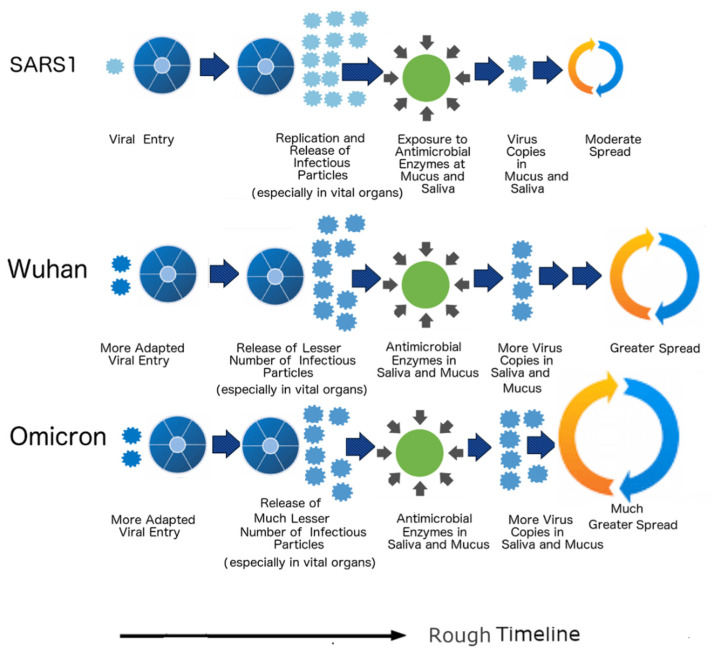
The life cycles of the SARS-CoV-1/2, SARS-CoV-1 (SARS1) produces more infectious particles, especially in vital organs arising from the differences in N PIDs but sheds fewer infectious particles orally and nasally because its outer shell (M) is not as hard and protective as that of SARS-CoV-2. SARS1 and SARS-CoV-2 have different life cycles as a result of the differences in the S proteins. This is denoted by the differences in the lengths of their life cycles and the number of particles at viral entry. Note: Numbers of infectious particles shown are comparative and qualitative illustrations and are not to meant to be exact quantifications.

In our previous studies, we have shown that the life cycles of SARS-CoV-2 variants are different from that of SARS-CoV-1. The lower disorder in N protein contributes to the fewer infectious particles produced in vital organs in contrast to the N of SARS-CoV-1. The abnormally low M disorder found in all COVID-19 CoVs protects the particles from the harsh antimicrobial enzymes and thus allows heavy nasal and oral shedding [16,17,32]. The S protein does affect the life cycle in different ways. The S protein of SARS-CoV-2 contains a polybasic furin cleavage site not found in SARS-CoV-1, pangolin-CoVs, or RaTG13 that assists in the more efficient viral entry [2,17,99]. It is for this reason that experimental studies have shown that SARS-CoV-2 produces more intracellular RNA than SARS-CoV-1, whereas SARS-CoV-1 produces more infectious particles than SARS-CoV-2 [100,101]. These differences are summarized in Figure 6, where we can see two viral particles entering the Wuhan-Hu-1 strain and Omicron variant as opposed to the single particle in SARS-CoV-1 (SARS1). The life cycle lengths of the Wuhan-Hu-1 strain and Omicron variant are also longer than that of SARS-CoV-1. Clinical studies have shown that SARS-CoV-2 sheds more particles from the beginning when the patients show the first sign of its symptoms until right to the end, with many patients remaining infectious even after the symptoms are gone [101,102]. In the case of SARS-CoV-1, shedding begins late and lasts only for a while. Omicron has a different life-cycle from the Wuhan-Hu-1 strain, showing its symptoms very quickly [12,13]. All these factors likely arise from the differences in the S protein–protein interactions.

## 4. Discussion

### 4.1. The Shell Disorder Approach to Solve the Mysteries of Omicron

We have seen that the SDMs have provided evidence that Omicron has a specific and peculiar nature in terms of evolution. The virulence–inner shell disorder model has identified it to be attenuated based on the Omicron low N-PID of 44.8%. In a previous study, the model had identified the 2017 pangolin CoV or a similar strain to be a potential attenuated precursor to the SARS-CoV-2 that may have been spreading before the pandemic [16]. The 2017 pangolin CoV has an N-PID of 44.9%, which is very close to that of Omicron.

As for the greater infectivity of Omicron, the transmission–shell disorder model points to its low M-PID, lower than that of all the SARS-CoV-2 variants including the Wuhan-Hu-1 strain. Just as SARS-CoV-2 is more contagious than SARS-CoV-1 due to the difference in M-PIDs, the same can be said of Omicron and the rest of the variants (Table 1 and Table 2, Figure 4C). The hard outer shell (low M-PID) facilitates greater protection against the antimicrobial enzymes found in the mucus and saliva [62,63]. As a result, the virus is able to shed large numbers of particles orally and nasally without overwhelming vital organs with higher viral loads.

### 4.2. Where Was Omicron Hiding All these Years? According to the Shell Disorder Models: Among a Specific Species of Burrowing Animals

Upon the discovery of Omicron by a group of South African doctors, the variant was shrouded in complete mystery; even today, despite the emergence of the results from clinical, experimental, and computational studies, many aspects of the Omicron mystery remain unanswered. For instance, it was believed that Omicron diverged from the Wuhan-Hu-1 strain before all known variants but it remains a mystery as to where it was hiding all along, as there has been no genetic trace previously detected [14]. One suggestion was that it had been hiding in an immunocompromised individual, e.g., an HIV patient. It was discovered that SARS-CoV-2 remains in HIV-positive patients for many months [103]. Another possibility is that the virus had been hiding in other animals, such as mice [104].

In this paper, a different approach is used to look at Omicron. SDMs show that a phylogenetic tree using M is the best possible phylogenetic approach, as the models had found M to be the most conserved among all COVID-19-related viruses. The M phylogenetic tree concurs with the S1 phylogenetic tree in the sense that we see an Omicron divergence from a parent lineage at an early stage, unlike the other variants. As for where Omicron was hiding, SDMs have some specific answers. As seen in the M phylogenetic trees in Figure 4B,C, the closest relatives of SARS-CoV-2 are pangolin CoVs, as pangolin CoVs might provide an evolutionary environment suitable for the maintenance of hard outer shell (low M-PID) arising from the habits of pangolins. Not only does the SDMs suggest that SARS-CoV-2 arose from pangolin-CoVs, it is likely that the virus has been moving in both directions between humans and pangolins as highlighted in blue (Figure 5). In fact, Figure 4B,C tell us that the pangolin CoVs are probably the closest relatives of Omicron.

As for hiding in the body of an immunocompromised individual [103], the fact that the M-PID of Omicron is lower than M-PIDs of all other SARS-CoV-2 variants seems to provide evidence that hiding in an immunocompromised patient probably did not happened, as it is difficult to envision that a harder outer shell would provide any evolutionary advantage in the body of such an individual. On the other hand, a lower M disorder does provide definite advantages in the body and buried feces of a burrowing animal as it helps facilitate fecal–oral–respiratory transmission as the virus needs to remain intact in buried feces for a long time before infecting the next host. The suggestion that the virus was hiding in mice, as mentioned in several studies [104], is, however, complex. Mice have two competing evolutionary pressures. Mice in the wild live in burrows, whereas those in urban settings and villages live in houses with humans [105]. Mice have evolved with humans for centuries. This complexity is also captured in our data on disorder analysis. While MHV (murine hepatitis virus) has a somewhat high M-PID of 8%, the closely related HCoV-HKU1 has one of the lowest M-PID values (4.2%) known (Table 1). There is still an air of mystery surrounding HCoV-HKU1, even if we know that it is closely related to MHV. We still have absolutely no knowledge on how or when the virus entered humans [59,60].

HCoV-HKU1 presents to us yet with another enigma that could be superficially used as a counter example to disprove the shell disorder models. The first human case was discovered in 2004 in a 71-year-old man. Ever since the discovery, it has been noticed that the occurrences of outbreaks have been sporadic [59,60]. A question is then: How can HCoV-HKU1 not be as infectious when its M PID is lower than that of SARS-CoV-1? The answer has to do with the fact that the N PID of HCoV-HKU1 is the lowest (N PID: 37%, see Table 1) seen in any CoVs. We have already seen that the N protein assists in the replication of the virus, such that its greater disorder helps the virus to produce sufficient virus copies necessary for respiratory transmission to be even viable. In the case of HCoV-HKU-1, its abnormally low N PID may be inhibiting its respiratory transmissibility, despite its extremely low M PID. It is for this reason that we believe that HCoV-HKU1 should remain in group C, not D, unlike other CoVs with similarly low M PIDs.

### 4.3. Omicron, Like Pangolin-CoV 2017, Is Inherently Attenuated

From the beginning, physicians in South African had observed that most Omicron patients had mild symptoms and did not require hospitalization or oxygen ventilators, unlike previous outbreaks. As more data arrived from South Africa and the rest of the world, the milder nature of Omicron was confirmed to be true [11,12,13,33,34]. Furthermore, experimental studies performed by various laboratories throughout the world using tissues or animal models have demonstrated that more viral particles can be found in nasal and bronchial tissues than those of lungs [33,34].

As we have seen, the virulence–inner disorder model did predict the Omicron attenuation. In fact, we also suggested in previous studies that pangolin CoVs may have quietly entered the human population around 2017 or before as an attenuated precursor of SARS-CoV-2. The prediction was based on the assumption that the precursor was something very similar to the 2017 pangolin CoV, which has M- and N-PIDs of 5.8% and 44.9% (Table 2). As aforementioned, we may notice that its N-PID is very similar to that of Omicron (44.8%), which implies that the SDMs have actually predicted Omicron to be attenuated even before its discovery. We also predicted that the precursor would spread more slowly than SARS-CoV-2. This is still to be true as we were assuming an M-PID of 5.9% or higher, unlike the Omicron M-PID of 5.4%. While Omicron has a harder outer shell; i.e., it is more capable of resisting the harsh nasal and mucosal enzymes, this may not be necessarily true for the predicted SARS-CoV-2 precursor that is believed to be attenuated. In fact, two of three known pangolin CoVs have M-PID values that are the same or higher than those of the Wuhan-Hu-1 isolate and Delta variant.

### 4.4. How Did It Reach South Africa?

As we are seeing, fast-spreading Omicron causes a larger than previous numbers of death, even when attenuated, because of the speed of the spread, resulting from its harder outer shell. This may not have been the case for a SARS-CoV-2 precursor, as it had silently and, perhaps, slowly but steadily spread in humans for several years before it became more virulent. If it was a slow-moving and attenuated virus, doctors could have easily mistaken it for a cold. While scientists have pinpointed the Wuhan Seafood market as the epicenter of an index case [106], this index case may have been a reflection of the initial virus mutation into a more virulent form, not that it was the index case of a first human spread with an attenuated precursor that had happened several years before 2019.

These considerations, too, may explain the enigma of how did Omicron reached South Africa so early, assuming that it was hiding in some burrowing animals in South Africa? One possible explanation is that by the time Omicron had undergone a reverse zoonotic transfer back to animals, the precursor virus had already silently spread to a large human population and had even spread overseas and reached South Africa via air travel. South Africa has a large variety of burrowing animals, including African pangolins [98]. It is not difficult to imagine a scenario in which the virus could re-enter the animal population through human activities. For instance, an infected person could dispose of his feces as trash along with sugary foods that attract ants, which then in turn attract pangolins, and the pangolins eventually eat the ants along with the virus-contaminated feces. This hypothesis is also supported by the phylogenetic tree using M, which reveals that the Omicron did not originate from the Wuhan-Hu-1 strain but instead from one of its ancestors that has a closer relationship to pangolin CoVs (Figure 4C). The reliability of this tree is supported by the evolutionarily conserved nature of the M, which involves transmission via buried feces, and the fact that current phylogenetic algorithms handle recombination very poorly [97].

### 4.5. Shell Disorder Predictions Are Consistent with Incoming Experimental and Clinical Data Pertaining to Omicron

SDMs present a unique explanation for Omicron attenuation, which has been confirmed clinically. Omicron is mild because its N protein is less disordered and therefore is unable to assist the virus more effectively in the “Trojan Horse” immune evasion strategy, in which the virus tries to replicate rapidly before the host immune system can detect its presence. As a result, fewer viral particles are produced in vital organs such as the lungs. On the other hand, increased oral and nasal shedding also occur because of Omicron’s harder outer shell that is more able to resist the antimicrobial enzymes found in the mucus and saliva. This mechanism is actually supported by experimental studies throughout the world. An example is the study performed by Hong Kong University (HKU) that involves the collection of tissues from the bronchi and lungs separately [34]. The tissues were then infected with Omicron, Delta, and the Wuhan-Hu-1 isolate, and the amounts of infectious particles present were studied and quantified. The bronchial tissues infected with Omicron were found to have 70 times more viral particles of the bronchial tissues infected with the Delta or the original variant, whereas the lung tissues infected with Omicron had 10 times fewer viral particles than the those infected with Wuhan-Hu-1. This is consistent with the predictions made by SDMs, since the walls of the bronchi and bronchial cells are lined with a layer of mucus, unlike the lung alveoli. Similar results have been obtained using mice and hamsters as animal models; again [33], they do not contradict the SDMs. There is also evidence that the layers of mucus in the bronchi act as a transport vehicle that moves foreign matters away from the lungs, which could ultimately increase the concentration of viral particles in the nasal cavity.

### 4.6. N and M Disorder Correlates with Viral Titers of SARS-CoV-2 in Lungs and Bronchi

If we look more closely at the data coming from HKU [34], something else intriguing can be seen. At 48 h after infection in lung tissues, tissues infected with the Omicron variant, the Delta variant, and the Wuhan-Hu-1 isolate had viral titers of 1 × 10^3^, 3 × 10^3^, and 9 × 10^3^ TCID50 per mL (the median tissue culture infectious dose, which is defined as the dilution of a virus required to infect 50% of a given cell culture). These were positively correlated (r = 0.88, *p* = 0.11; the poor p-value is due to small sample size, which is inevitable in this case) with the N-PIDs of 44.8%, 47.1 ± 0.6%, and 48.2%, respectively (Table 1 and Table 2). Infected bronchial tissues yielded even more interesting results. These tissues infected with the Omicron variant, Delta variant, and the original SARS-CoV-2 strain yielded viral titers of 6 × 10^4^, 1 × 10^4^, and 1 × 10^3^ TCID50 per mL. While the yield from the Omicron and Wuhan-Hu-1 infections can be explained by the hardness of the M (outer shell) of 5.4% and 5.9%, the yield of Delta is a puzzle since it has the same M-PID as the Wuhan-Hu-1 isolate. One explanation may involve the hardness of the inner shell, N. We need to remember that the Delta variant has slightly lower N-PID of 47.1 ± 0.6% than the wild-type N-PID of 48.2% (strong negative correlation of bronchial viral titer to N- and M-PIDs (r = 0.99, *p* = 0.07, poor *p*-value due to small sample size). This basically implies that the inner shell also plays some role in protecting the virion, namely the RNA, from damaging effects of the antimicrobial enzymes found in the mucus.

### 4.7. Negative Correlation of Viral Titer with Shell Disorder in the Bronchi, Not Lungs: Mucus Network in Bronchi, Not Lung Aveoli

An important question is: Why does the viral titer of infected lung tissue positively correlate with the N disorder, whereas the viral titer of the infected bronchial tissues have negative correlation with N and especially M disorder? The answer is that there is a presence of mucus in walls and cells of the bronchi that is not found in the lung aveoli [107,108]. Not only does the mucus in the bronchi have antimicrobial enzymes that could damage the virus [62,63], the mucus network in the wall of the bronchi acts to transport viruses and other harmful objects, which it is unable to destroy, away from the lungs [107,108]. The lungs, on the other hand, can be divided into the conducting and respiratory zones [108]. The conducting zone includes the bronchioles that, as mentioned, use mucus to transport foreign materials away from the lungs. More importantly, there is also the respiratory zone that includes alveolar cells that are not covered by mucus, but rather by a surfactant [107] that also acts to remove foreign debris away from the lungs. In other words, much of the lung is devoid of mucosal antimicrobial enzymes, unlike the bronchi and bronchial cells. This is a highly crucial factor that has unfortunately been totally overlooked in all current analyses of such experiments, lead to many misconceptions of the actual mechanism of viral infections involved.

### 4.8. New Knowledge from SARS-CoV-2 and Omicron Is Strengthening Our Understanding of the Shell Disorder Models as Applied to SARS-CoV-2

We have previously seen the characteristics of both inner and outer shells protecting the virion from damage, such as in the case of the rabies virus [15,23,24] and the close retroviral relatives, HIV and EIAV [25,27]. HIV is predominantly sexually transmitted and does not have to be exposed to any harsh environment, whereas its horse cousin, EIAV, is transmitted by a blood-sucking horsefly that stores infected blood mixed with its saliva in its mouthpiece. It is therefore also not surprising that while HIV-1 has a high outer shell disorder (matrix PID: 56.5 ± 10.8) and a moderately high inner shell disorder (capsid PID: 44.6 ± 2.8; nucleocapsid: 39.5 ± 3), EIAV has very low PIDs for all its shell proteins (matrix PID: 13 ± 0.1; capsid PID: 29 ± 0.1; nucleocapsid PID: 26 ± 0.1%). What is amazing is the fact that Omicron does not only reproduce what the SDMs are again predicting, but also increases our knowledge relating to the models. We also know now that pangolins provide evolutionary advantages for lower N-PID, which protects the virus in buried feces. This is an added reason to believe that Omicron may have been hiding in a burrowing animal, such as a pangolin, for many years. The burrowing animal provides an ideal evolutionary environment for lower N and M disorder.

## 5. Summary and Conclusions

While Omicron is currently shrouded with mysteries, SDMs have specific answers to many of these mysteries that are linked to the peculiar evolution of SARS-CoV-2 in general, and not just Omicron. Prior to the discovery of Omicron in South Africa, the SDMs suggested that an attenuated precursor may have entered humans from pangolins in 2017, if not earlier, and that this precursor was spreading more slowly than the SARS-CoV-2 (Wuhan-Hu-1). This prediction was based on the lower N disorder levels (N-PID: 44.9%) with the assumption that the M remains the same or is higher than that of Wuhan-Hu-1. When the fast-moving but milder Omicron emerged, it was revealed that the N of Omicron resembles pangolin CoV 2017 (N-PID: 44.8%), but that this variant showed harder outer shell (M-PID: 5.4%). We were unsurprised by the attenuation, but were surprised by the effects of a harder outer shell, even when we already knew that SARS-CoV-2 was more contagious than SARS-CoV-1 (M-PID: 9%) because of the much harder outer shell of SARS-CoV-2 (M-PID: 5.9%). SARS-CoV-1 is more virulent than SARS-CoV-2, as SARS-CoV-1 has a greater disorder in its inner shell protein (N-PID: 50%) compared to SARS-CoV-2 (Wuhan-Hu-1 N-PID: 48.2%), since the virulence–inner shell disorder model suggests that greater disorder at the inner shell helps faster replication of viral particles, especially in vital organs. SARS-CoV-2, on the other hand, sheds more infectious particles because the harder outer shell protects the virion from harsh environments and oral and nasal antimicrobial enzymes.

This paradigm is consistent with the incoming data pertaining to Omicron. For instance, using incoming data from HKU, strong correlation is found between N-PID and viral titers of lung tissues infected with Omicron, the Wuhan-Hu-1 strain, and Delta, whereas, in the case of bronchial tissues, correlation is found between shell (N- and M-PID) hardness and viral titers. What is surprising is that Omicron and Delta indicate that both shells play roles in protecting the virion from damage. We have actually seen this characteristic in other viruses, such as EIAV. Nevertheless, Omicron has shown us that the outer shell hardness contributes the most towards the infectiousness of the disease. Once again, the COVID-19 pandemic is an event that is not only reproducing our predictions, but also adding to our knowledge on how SDMs should be applied more accurately to SARS-CoV-2 and perhaps even other viruses.

We have seen that the outer shell plays an important role in the spread of CoV in both humans and pangolins. The hard outer shell (low M-PID) allows greater contagiousness, whereas the low M disorder provides greater fitness in the fecal–oral–respiratory spread among burrowing animals, such as pangolins, likely via buried feces. As a result, M-PIDs of all COVID-19-related CoVs are among the lowest in the entire CoV family, making it ideal for the phylogenetic study as it is evolutionarily conserved. Indeed, the phylogenetic tree using M has uncovered many things not observed before. It revealed that Omicron did not split from the Wuhan-Hu-1 strain, but instead from one of its ancestors that was closer to pangolin CoVs. This raises many questions; we have already addressed some of them. A previous phylogenetic study using S1 mentioned that Omicron broke off from the original strain a few months after the first outbreak that began at the end of 2019. We believe that such studies have been misdirected by the high probability that Omicron had acquired bits and pieces of proteins from other variants when it re-emerged from a burrowing animals back to humans. Current phylogenetic algorithms do not handle recombination well [97]. The SDMs also suggest that Omicron had been hiding among a species of burrowing animals, such as pangolins, through reverse zoonotic transfer back to animals, as burrowing animals would provide the necessary evolutionary environment for the virus to retain its attenuation and still increase the hardness of its outer shell as a result of the behaviors of such animals via exposure to buried feces. The Omicron data add important evidence to the suggestion that an attenuated SARS-CoV-2 precursor entered humans in 2017 or earlier from pangolin-CoVs.

## Data Availability

Not applicable.
